# Flexibility and extracellular opening determine the interaction between ligands and insect sulfakinin receptors

**DOI:** 10.1038/srep12627

**Published:** 2015-08-12

**Authors:** Na Yu, Moises João Zotti, Freja Scheys, Antônio S. K. Braz, Pedro H. C. Penna, Ronald J. Nachman, Guy Smagghe

**Affiliations:** 1 Department of Crop Protection, Faculty of Bioscience Engineering, Ghent University, 9000 Ghent, Belgium; 2 Molecular Entomology and Applied Bioinformatics, Department of Crop Protection, Federal University of Pelotas, 96010-900, Pelotas, RS, Brazil; 3 Laboratory of Computational Biology and Bioinformatics, Federal University of ABC, 09210-170 Santo André, Brazil; 4 Insect Control and Cotton Disease Research Unit, Southern Plains Agricultural Research Center, USDA, College Station, TX 77845, USA

## Abstract

Despite their fundamental importance for growth, the mechanisms that regulate food intake are poorly understood. Our previous work demonstrated that insect sulfakinin (SK) signaling is involved in inhibiting feeding in an important model and pest insect, the red flour beetle *Tribolium castaneum*. Because the interaction of SK peptide and SK receptors (SKR) initiates the SK signaling, we have special interest on the structural factors that influence the SK-SKR interaction. First, the three-dimensional structures of the two *T. castaneum* SKRs (TcSKR1 and TcSKR2) were generated from molecular modeling and they displayed significance in terms of the outer opening of the cavity and protein flexibility. TcSKR1 contained a larger outer opening of the cavity than that in TcSKR2, which allows ligands a deep access into the cavity through cell membrane. Second, normal mode analysis revealed that TcSKR1 was more flexible than TcSKR2 during receptor-ligand interaction. Third, the sulfated SK (sSK) and sSK-related peptides were more potent than the nonsulfated SK, suggesting the importance of the sulfate moiety.

Insect sulfakinin (SK) signaling is active in a variety of biological processes in insects, such as the regulation of feeding^1^,^2^,^3^,^4^,^5^ and muscle contraction^6^,^7^,^8^. SK signaling consists of SK peptides, SK receptors (SKRs) and other molecules. The interaction of SK peptides and SKRs initiates the SK signaling transduction. SK peptides share a conserved carboxyl-terminal amino acid sequence YGHMRF-NH_2_ with different amino-terminal extensions^9^. Two forms of SK peptides are often found in insects as sulfated SK (sSK) and nonsulfated SK (nsSK), depending on the presence of a sulfate group on the tyrosyl residue^8^. SKRs are G-protein coupled receptors (GPCRs) that can convert the extracellular signals into intracellular signals^10^.

Insect SK signaling is found to be homologous to the cholecystokinin (CCK) signaling in humans since they show homology over their components, signaling transduction and functions^2^,^6^,^7^,^9^,^11^,^12^. Human CCK signaling has been investigated extensively. It involves the CCK peptides and CCK receptors^13^. CCK peptides exist in two forms: sulfated CCK (sCCK) and nonsulfated CCK (nsCCK). In human CCK signaling, two CCK receptors (CCKRs), namely CCK1R and CCK2R, show different affinities to sCCK and nsCCK. CCK1R is activated by sCCK 500- to 1000-fold more than by nsCCK, while CCK2R responds to both sCCK and nsCCK similarly^13^. The ligand binding sites in CCKRs have been examined via site-directed mutagenesis^14^,^15^,^16^,^17^,^18^, photoaffinity labeling^19^,^20^ and molecular modeling^16^,^17^,^18^,^19^. Several amino acid residues demonstrate their pivotal role in the interaction of CCK and CCKR, such as the Met195 and Arg197 in the second extracellular loop^17^,^18^. However, little is known about the insect SK signaling in terms of the interaction of ligand and receptors.

As a satiety factor, insect SK signaling provides insights into the development of new pest control strategies. Our previous experiments showed that in the important model and pest insect, the red flour beetle *Tribolium castaneum*, sSK peptide is 1,000–10,000 times more potent than nsSK to activate the two SKRs (TcSKR1 and TcSKR2)^21^, and silencing of two TcSKR genes resulted in altered feeding behavior to different extent^4^,^5^. This lead to a hypothesis that sSK and nsSK interact with the two TcSKRs differently. Therefore, understanding the interaction of ligand and receptor will promote the application of SK signaling in both fundamental research and pest management. SK peptides and SK receptors are the two parties that interact with each other and their intrinsic properties affect the interaction. The most notable properties of SK peptides are the amino acid composition and the sulfate moiety and the ones of SKRs are their complex structures. Therefore, an *in vitro* SKR activation bioassay was conducted with a series of SK-related peptides to assess the factors from the SK peptide. Molecular modeling and normal mode analysis (NMA) were carried out to realize the three-dimensional structure of the two SKRs, aiming at the structural features that may affect the interaction, where special attention was given to the outer opening and flexibility of SKR proteins.

## Results

### TcSKR1 and TcSKR2 contained cavities with different outer opening

**Figure 1:**
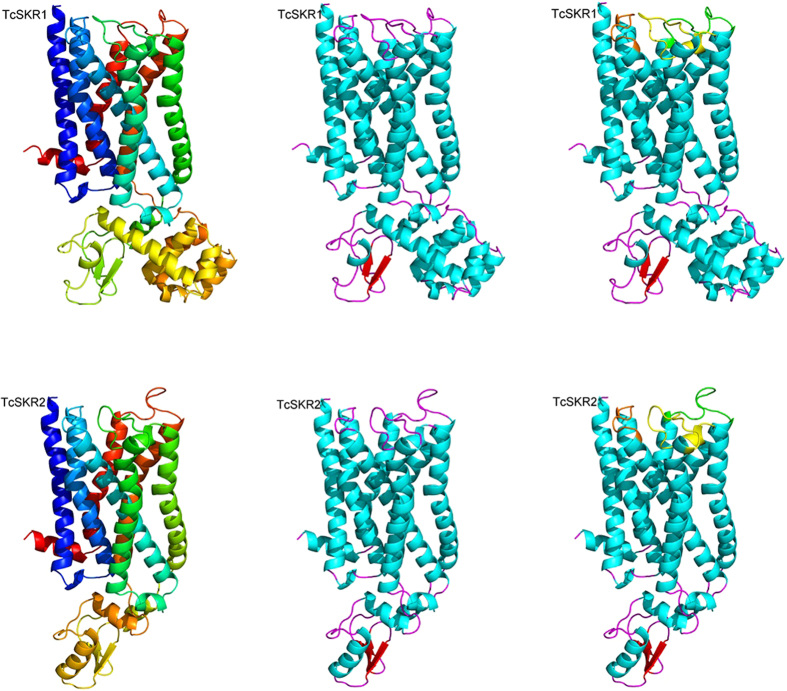
Cartoon diagram of *Tribolium castaneum* sulfakinin receptor 1 (TcSKR1) and *T. castaneum* sulfakinin receptor 2 (TcSKR2). The seven transmembrane α-helices building the three-dimensional fold of the proteins are differently colored from blue (N-terminus) to red (C-terminus) (upper and lower on the left) by secondary structure (upper and lower in the middle) and with extracellular loops (ECL) colored differently (upper and lower on the right). The ECL 1 is colored in orange, ECL 2 in yellow and ECL 3 in green. The N-terminus is colored in blue while C-terminus red.

TcSKR1 and TcSKR2 both consisted of the canonical seven α-helices crossing the cell membrane as commonly found in GPCRs (Fig. 1). Both TcSKRs exhibited three extracellular loops (ECLs) with similar number of residues. Remarkably, TcSKR1 showed eight intracellular small α-helices and two β-sheets, while SKR2 had three small α-helices and also two β-sheets.

**Figure 2:**
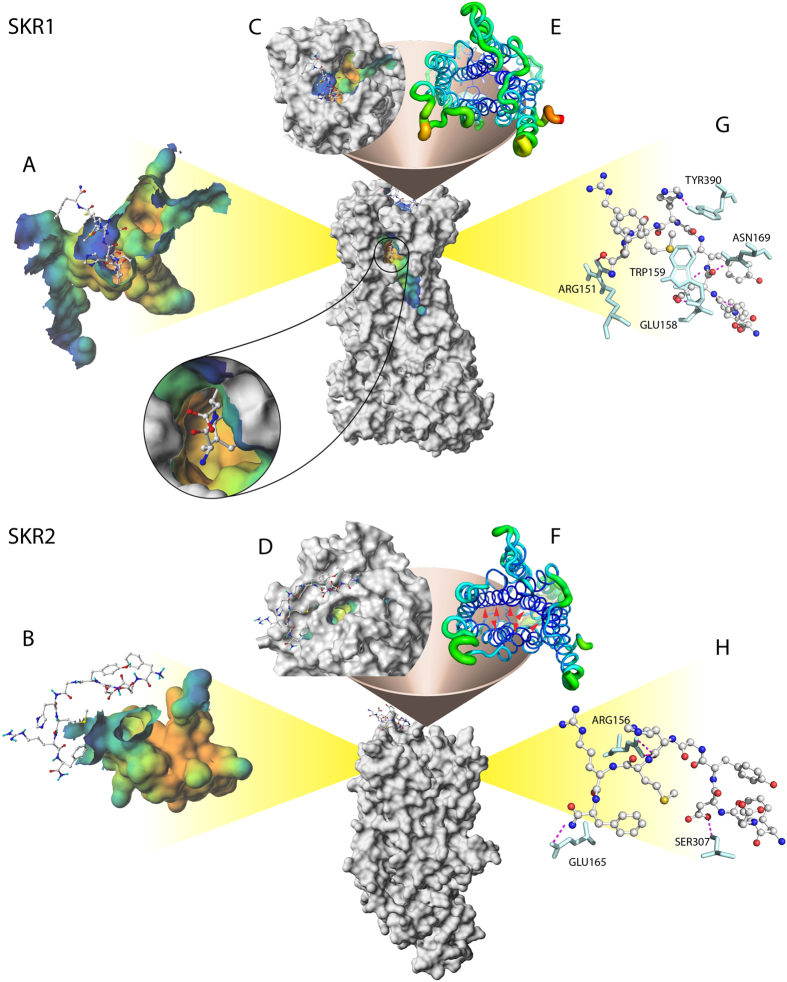
Surface, ribbon, flexibility and interaction diagrams generated by normal mode analysis of TcSKR1 (**A**,**C**,**E**,**G**) and TcSKR2 (**B**,**D**,**F**,**H**) with nonsulfated sulfakinin (nsSK) peptide docked (colored by atom type). Cavities are colored by depth from blue (shallow) to orange (deep) in TcSKR1 (**A**) and TcSKR2 (**B**). The flexibility for each amino in SKR1 and SKR2 was retrieved from normal model analysis and the root-mean-square fluctuation (RMSF) was plotted onto each structure and colored from dark blue (rigid) to red (highly flexible) (**E**,**F**). The picture demonstrates a deep cavity in both structures but with an extended opening out cavity in SKR1 (**C**). The red arrows (**F**) indicate the most significant region responsible for reduction in outer openings of SKR2 cavity (**D**). The top view of TcSKR2 (**D**,**F**) displays a rigid core that restricts a larger outer opening of the cavity, which to some extend hinders a deeper intrusion of peptides into cavity. The figure C shows the larger outer opening of SKR1 allows the peptides to go deeper into cavity (see lupe detail). Docking of nsSK to TcSKR1 (**C**) and TcSKR2 (**F**). The clips show the binding of nsSK in both receptors through several hydrogen bonds colored by pink dashed lines. The residues are indicated by names and colored as light cyan.

Figure 2 depicts the largest channel found in each modeled receptor that was calculated by MOLCAD. The cavities of both TcSKRs were similar in deepness from blue (shallow) to orange (deep). However, TcSKR2 had a narrow outer opening that prevented a deep intrusion of ligands into protein volume (Fig. 2D). In contrast, TcSKR1 exhibited a cavity similar in depth, but with a much larger outer opening than TcSKR2 (Fig. 2C).

### TcSKR1 was more flexible than TcSKR2

As demonstrated in Fig. 2, the cavities of TcSKR1 and TcSKR2 were similar in deepness; however, the size of their outer opening exhibited a substantial difference (Fig. 2C,D). In this case, the deepness of the cavity in TcSKR2 (Fig. 2B) was considered to be irrelevant for peptide activity since the ligands did not have a deep access into TcSKR2. TcSKR2 displayed an extremely rigid core structure that prevented large movements during receptor-ligand interaction (Fig. 2F). On the other hand, TcSKR1 displayed a much looser core structure that allowed free movements and therefore a cavity with a larger outer opening (Fig. 2C,E). This opening provided an enter access responsible for harboring ligands such as peptides. The comparatively higher flexibility observed in TcSKR1 allowed nsSK to go deeper into the cavity (Fig. 2; see lupe detail).

**Figure 3:**
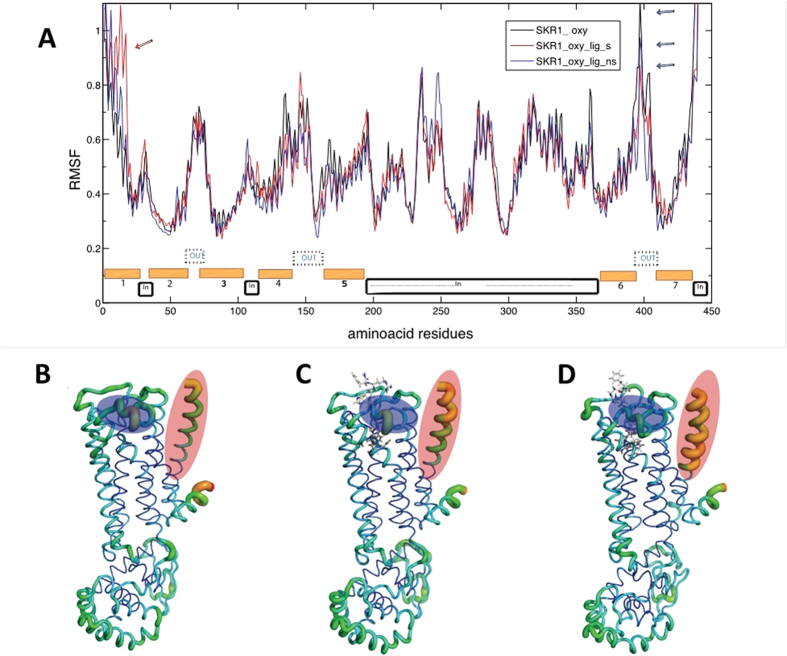
Flexibility demonstrated by a graph constructed using root-mean-square fluctuation (RMSF) for each amino acid in TcSKR1 model (**A**) and RMSF plotted onto TcSKR1 structure (**B**–**D**). The corresponding amino acids for TcSKR1 are indicated along with the x-axis. The calculations were performed with empty cavity of TcSKR1 in oxidized state (SKR1_oxy, black line), with docked nonsulfated sulfakinin (SKR1_oxy_lig_ns, blue line) and with docked sulfated sulfakinin (SKR1_oxy_lig_s, red line) and the retrieved flexibility (RMSF) was plotted onto structures (**B**–**D**), respectively. The transparent ellipses indicate regions with remarkable changes. The red ellipse corresponds to transmembrane region I and blue ellipse corresponds to extracellular loop 3. These regions are also indicated in the line graph by a red arrow or blue arrows.

In the present project, we also used NMA to investigate structural variation in the flexibility of TcSKR1 imposed by sSK and nsSK. The two forms of SK introduced specific variations at very much defined regions (Fig. 3A). Transmembrane I (TM I, red ellipse in Fig. 3) showed a steadily increase in flexibility from receptor with empty cavity, with docked nsSK and with docked sSK (Fig. 3B–D, respectively). sSK introduced a considerable stabilization in ECL 3 (Fig. 3D). The ECLs (blue ellipse in Fig. 3) showed medium flexibility in receptor with empty cavity, decreased the flexibility in receptor with docked nsSK, and became almost rigid in the receptor with docked sSK (Fig. 3B–D, respectively). Both receptor models were stable during 200 ns of molecular dynamics, with variation of backbone (RMDS) around 0.3 nm (Supplementary figure 1). Nevertheless we noted that SKR1 is more stable when peptides occupy the cavity.

### Molecular modeling of the SK-SKR complexes

**Figure 4:**
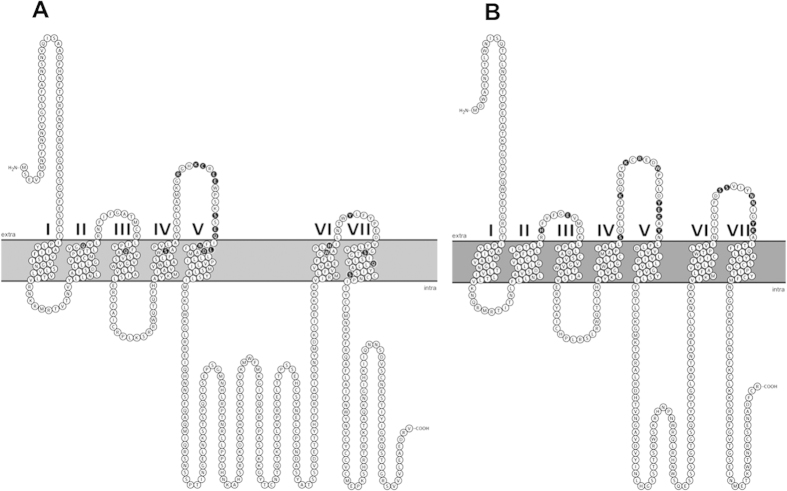
Amino acid residues involved in binding of SK-related peptides in TcSKR1 (**A**) **and TcSKR2** (**B**). The residues were collected for the binding of TcSKRs with sSK, nsSK, alanine-substituted and truncated SK peptides in Table 1 and Table supplement 1. The residues are represented with white single-letter codes in black filled circles. Figures were generated online with Protter v.1.0.

Figure 4 summarizes the amino acid residues involved in the binding of TcSKRs and SK-related peptides tested in Table 1. The interactive residues in receptors were found in both the extracellular region and transmembrane (TM) region in TcSKR1, while they were only located in the extracellular region in TcSKR2 (Fig. 4; supplementary table 1). For these docked peptides, with exception of S143 of TM 4, the TM regions in TcSKR2 were not involved in the ligand-receptor interaction.

### Sulfate moiety affected the binding of SK to SKR

Sulfated SK (sSK) and nonsulfated SK (nsSK) were docked in both TcSKR1 and TcSKR2. The interactive residues from both peptide and receptor are summarized in Table 2. First, in the TcSKR1-SK complexes, four sSK residues interacted with five TcSKR1 residues and five nsSK residues interacted with five TcSKR1 residues with three identical interactions. In the TcSKR2-SK complexes, three different residues in sSK and nsSK interacted with three TcSKR2 residues, respectively. Second, in the TcSKR1-SK complexes, the sulfated tyrosine (Y6) in sSK interacted with both D221 and E206, while nonsulfated tyrosine in nsSK interacted with E206 only. In contrast, in the TcSKR2-SK complexes, the tyrosine was inactive in the binding to TcSKR2 regardless the presence of a sulfate group. Lastly, as a general observation, the interactive residues were found in mainly the extracellular regions in TcSKR2, but in both the extracellular regions and the transmembrane regions in TcSKR1.

### Peptides with alanine-substitution were posed differently to TcSKR1 and TcSKR2

The peptides 2003–2008 with alanine-substitution of nsSK were docked to both TcSKRs. The interactive residues are presented in Table supplement 1. A general finding was that these peptides made contact with TcSKR1 differently from how nsSK did, whereas they interacted with TcSKR2 in a similar manner as nsSK did. NsSK bound to TcSKR1 via the ECL 2 and the TM V and VI. NsSK bound to TcSKR2 via the ECL 2 and the ECL 3. Peptides 2003–2008 bound to TcSKR2 via the same regions and the ECL 3 was also recruited.

### Composition of SK peptide affected the SK-SKR interaction

Transiently transformed insect cells were used to assess the activity of a series of SK-related peptides on the two individual TcSKRs. The relative bioactivity of tested SK-related peptides on the two TcSKRs is shown in Table 1. The peptides all resulted in the typical sigmoid dose-response curves and reached the maximal activity at 10 μM. First, peptides 1835, 1591-1 and 1658 are sulfated peptides with similar amino acids as sSK. These sulfated SK-related peptides were much more active than nsSK on both TcSKRs. Second, peptides 2003–2008 contain an alanine substitution of one single amino acid within nsSK, and peptides 2009–2076 are truncated versions of nsSK peptide. None of these peptides were active when compared to their parental peptide nsSK, which suggests that the alanine substitution is fatal to the nsSK and the length of nine amino acids is essential for the interaction of SK and SKR.

Single substitution of nsSK led to inactive peptides to TcSKRs such as peptide 2003 (F → A), 2004 (R → A), 2005 (M → A), 2006 (H → A) and 2008 (Y → A). These alanine-substituted nsSK-related peptides may have lost their activity due to the simple structure of A, as amino acids F, R, M, H and Y all feature a specialized and larger functional group on the side chain. Peptide 2007 (G → A) kept the activity on TcSKR1 to some extent, which may be because of the similar structure and flexibility of glycine and alanine. Other substitutions resulted in peptides with retention of activity. These were peptides 1835 (F → S), 1658 (M → Nle) and 1591-1 (R → K). These substitutions were mostly with amino acids of similar properties to a certain extent. Therefore, the right property of amino acid content was important to TcSKR1. The removal of amino acids from the N-terminus of nsSK led to a drastic loss of activity (Table 1). This suggests that the minimal length of nsSK for activating TcSKRs is FDDYGHMRF-NH_2_. Taken together, not only the sulfate moiety but also the right amino acid property contributes to the activity of SK.

## Discussion

The modeling of the two TcSKRs revealed their structural characteristics for the first time and confirmed their property of being GPCRs. Very interesting is the difference of the outer opening between TcSKR1 and TcSKR2. By far, our studies showed that the two TcSKRs may respond differently to SK-related peptides and be involved in different biological processes^4^,^5^,^21^. The detailed information on their three-dimensional structures may provide clues to interpret their potential different roles.

In the previous experiments^21^, TcSKR1 was more sensitive to sSK than TcSKR2. The structural property of the TcSKR-sSK complex provides some clues to decipher this observation. Here, the predicted TcSKR1 model exhibited a larger outer opening of the cavity compared to the TcSKR2 model. This larger opening allowed peptides to be docked deeper into the cavity, differently from what occurred in TcSKR2 where peptide could not be docked into protein volume. Therefore, all peptides were laid on the top of receptor in TcSKR2-ligand complexes (Fig. 2D), while in TcSKR1-ligand complexes, peptides were posed in the receptor cavity (Fig. 2C). The consequence of these ‘lay-docked’ poses in TcSKR2-peptide complex was a close contact with the ECLs. The in-depth penetration of ligands into TcSKR1 created a lining of amino acids surrounding peptides in the cavity, which in turn provides a tight interaction through a network of hydrogen bonding.

The sulfate moiety on tyrosine has been reported to be important for the activity of SK, which was also observed in the cell-based receptor activation assay (Table 1). Based on the modeled SK-SKR complexes, the (SO_3_H) on tyrosine enhanced the binding of sSK and TcSKR1 via a TM residues (D221 in TM V), which contributes to the higher activity observed for sSK over nsSK (Table 2). Conversely, tyrosine in both sSK and nsSK made no contact with TcSKR2.

The ligand-receptor complexes showed some insights to explain the observed bioactivity of tested peptides, for example, the lack of activity in the alanine-substitution series. The amidated F of nsSK interacted with R199 in the ECL 2 of TcSKR1 in the TcSKR1-nsSK complex. In peptide 2003, this F is replaced by A. This modification generated more polar contact of TcSKR1 with peptide 2003; however, the hydrogen bond formed with R199 was lost. Archer-Lahoul *et al.*^22^ conducted several site-directed mutation experiments in CCKR1 coupled with docking of CCK. The replacement of R197 (equivalent to R199 in TcSKR1) by M197 caused a 3154-fold reduction in the affinity of CCK to CCK1R. Therefore, we suggest that the lack of interaction with this particular amino acid could abolish the binding affinity of peptide 2003 to TcSKR1. In the remaining alanine-substituted peptides (2004, 2005, 2006, 2007 and 2008), a similar number of hydrogen bounds was found but not any interacting with the alanine seemed crucial for receptor activation (Supplementary table 1).

Molecular modeling and docking are used to provide substantial structural knowledge about receptor-ligand complexes, from which functional information could be inferred. The modeling of ligand-receptor complex revealed the amino acid sites in both SK peptide and receptors that may be involved in their interaction. These sites will provide useful information for further investigation, such as being candidates for the site-directed mutagenesis study of receptor protein.

The NMA was used to investigate the flexibility changes of TcSKRs in an attempt to get insights that could explain the remarkable differences in the outer openings of the cavity. To our knowledge, this is the first time that such investigation has been conducted. The normal mode vibrations are harmonic oscillations characterizing the dynamics of the system around a minimum energy. In this case, each mode describes a state of the system where all particles are oscillating with the same characteristic frequency^23^,^24^. The NMA technique is based on a quadratic approximation of the potential energy surface and the equation of motion is solved analytically and its results are obtained in terms of directions of movements with their respective frequencies. This analysis provides a fair set of possible kinds of motion in TcSKRs.

The extracellular loops, named OUT regions in TcSKR1, were notably more flexible in reduced state and comparatively with TcSKR2 (Figure supplement 3 and Figure supplement 6). At least for the ECL 2 in TcSKR1, the presence of a cysteine may be responsible for that. This cysteine as well as its disulfide bond is conserved in most GPCRs^25^. Disulfide bonds formed by close cysteines in the extracellular domains of GPCRs are thought to be important to maintain the conformational integrity of the receptor, and in particular to allow ligand to access to the binding pocket^26^. In a biological context, these disulfide bounds are formed by oxidation of sulfhydryl groups (-SH). For NMA in an oxidized state, the cysteine located in ECL 2 is positioned close to another cysteine in top of TM III, forming a disulfide bond, which is expected to provide an additional structural constraint that contributes to the stability and conformation of the receptor. However, in the reduced state this interaction disappears and in turn this allows more loose movements (Figure supplement 3). sSK produced an increase of 1000 times in activity towards TcSKR1 compared to nsSK^21^. These differences can be correlated with specific variation in protein flexibility imposed by the sulfate moiety in sSK. While some regions such as TM I displayed increase and spreading in range of flexibility, other regions such as ECL 3 were stabilized. Therefore, further investigation on the mechanism is required.

In conclusion, the modeling of TcSKRs and ligand-receptor complex is indicative to the study of SK signaling in insects. TcSKR1 and TcSKR2 displayed similar GPCR characteristics but featured a different outer opening of the cavity, which affected the binding of TcSKRs with ligand peptide. TcSKR1 exhibited a more flexible structure than TcSKR2. The sulfate moiety on tyrosyl residue contributed to the higher activity of sSK over nsSK by enhancing the binding of sSK to TcSKR1.

## Material and Methods

### Comparative protein modeling and molecular dynamic simulations

The TcSKR1 and TcSKR2 models were generated using HHpred (http://toolkit.tuebingen.mpg.de/hhpred) for template identification, and MODELLER v9.14 for building the models. Both models were built based on human M2 muscarinic acetylcholine receptor (PDB: 3uon; identity: 23% and 21%). The stereochemical quality was evaluated utilizing the PROCHECK program^27^. More than 95% and 96% of the residues of the modeled TcSKR1 and TcSKR2, respectively, were correctly assigned on the best allowed regions of the Ramachandran plot, and the remaining residues were located in the marginal regions of the plot (Supplementary figure 2).

The complete system (protein, peptide, membrane, waters and boundaries) was assembled in a periodic rectangular box using Charmm-GUI membrane builder^28^ (http://www.charmm-gui.org/?doc=input/membrane). Each protein was inserted into a pre-equilibrated POPC (1-Palmitoyl-2-oleoylphosphatidylcholine) lipid bilayer with a hole whose diameter was comparable to the one found in TcSKRs (Supplementary figure 1). Molecular dynamic calculations were made in Gromacs 5.0^29^ (http://www.gromacs.org/), in which the system input was subject for energy minimization, equilibration and dynamic production using default settings. The only modification was to increase the dynamic production time to 120 ns.

### Flexibility study of TcSKRs

In this work, we used a normal mode analysis (NMA) to study the flexibility changes in the backbone structures of the two TcSKRs (TcSKR1 and TcSKR2) and in response to the two forms of SK peptide, namely sSK and nsSK. The flexibility was tested for TcSKR1 and TcSKR2 with empty cavity, and for TcSKR1 also with docked sSK and nsSK. Due to the amount of cysteine in both models, the NMA was carried out in the oxidized and reduced states. The disulfide bridge formed by the oxidation of two cysteine amino acids can be responsible for holding the proteins in their respective conformations and so influence NMA.

The proteins models generated from strategy described in comparative modeling and peptides were prepared using the CHARMM-GUI web server (http://www.charmm-gui.org). We minimized all these structures in vacuum using the CHARMM software with the force field charmm27 to prepare them for normal mode calculations. The all-atom normal modes of receptors and ligand combination were calculated using the CHARMM program. We calculated the first 27 internal lowest frequency modes of the minimized structures. These first modes describe the most representative global conformational changes for the molecule^30^. The root mean square fluctuations (RMSF) of Cα atoms and the average values per residue were calculated for each receptor and peptide combinations using CHARMM.

### Molecular modeling of ligand-receptor complex

Rigid molecular docking of the SK-related peptides (Table 1) into TcSKRs was performed with OEDocking suite for OSX (version 3.0.1; OpenEye, Santa Fe, NM) and flexible docking using FlexiDock from SYBYL. Several conformations were generated for each peptide with local minimum energies using OMEGA^31^ (version 2.5.1.4; OpenEye) force field mmff94 and root-mean-square-deviation (RMSD) set as 1.0. These conformers were docked separately in the TcSKR1 and TcSKR2 extracellular regions using FRED interface of OEDocking. In this first run of docking, the protein was kept rigid while an exhaustive search was applied for peptides. The exhaustive search systematically searched rotations and translations of each peptide conformer within the selected region.

The generated docking poses were ranked by each of the four individual scores (shape, hydrogen bound, protein desolvation and ligand desolvation). The sum of the normalized scoring functions generated the FRED Chemgauss4 score. The best-ranked pose of each peptide was inspected for steric hindrance. Those protein residues in close contact with the ligand or considered in potential for clashes were assigned as flexible for a new run of docking using FlexiDock. This last procedure was performed individually and continually for each peptide until the absence of clashes.

### Cells and peptide preparation

Sf9 cells (*Spodoptera frugiperda*) and Sf9-TcSKR1 and Sf9-TcSKR2 cells generated previously^21^ were routinely cultured in SF-900™ II SFM medium (Life Technologies, Merelbeke, Belgium) at 27 °C in darkness. The medium for Sf9-TcSKR1 and Sf9-TcSKR2 was supplemented with 20 μg/ml of puromycin (Life Technologies, Merelbeke, Belgium) to maintain the stable expression of TcSKRs.

SK-related peptides in Table 1 were designed based on the amino acid sequence of the natural SK peptide with either substitution or truncation. They were synthesized under previously described conditions^6^,^7^. The identity of the peptides was confirmed via matrix-assisted laser desorption ionization time-of flight mass spectrometry and quantified via amino acid analysis. Peptides were dissolved in stock solutions with 80% acetonitrile (in water). Working solutions were freshly prepared with cell culture medium prior to each experiment.

### Receptor activation bioassay

The reporter vector pGL4.29[*luc2P*/CRE/Hygro] (CRE; Promega, Leiden, the Netherlands) was applied here and it has a cAMP (cyclic adenosine 3′, 5′-cyclic monophosphate) response element upstream the reporter gene *luciferase*, through which the cAMP signaling regulates the transcription of *luciferase* proportionally. Thus, the readout of the luminescence corresponds to the extent to which the expressed TcSKR is activated in Sf9 cells. The SK-related peptides (Table 1) were tested at concentrations of 0.1, 1 and 10 μM.

Sf9, Sf9-TcSKR1 and Sf9-TcSKR2 cells were all transiently transfected with pGL4.29[*luc2P*/CRE/Hygro] using ESCORT™ IV transfection reagent (Sigma-Aldrich, Bornem, Belgium) following the manufacturer’s instruction. Medium was supplemented with 200 μg/ml hygromycin (Life Technologies, Merelbeke, Belgium) 24 h post-transfection. The transient cells (Sf9-CRE, Sf9-TcSKR1-CRE, Sf9-TcSKR2-CRE) were seeded at 20,000 cells per well in white 96-well plates (Greiner Bio-One, Frickenhausen, Germany). After 12 h, cells had attached to the well bottom and the medium was replaced by fresh medium containing peptide of designated concentrations. Each concentration was tested in three replicates and the entire assay was repeated at least twice. Two kinds of negative control were included. First, cell medium was used as a negative control reagent to give the background luminescence, given the solvent acetonitrile at the same final concentration in the cell culture medium demonstrated no effect on cell response. Second, Sf9-CRE cells were used as a negative control to eliminate the potential internal factors that were activated by peptide samples. Cells were incubated with a peptide for 5 h at 27 °C and then Bright-Glo™ luciferase assay (Promega, Leiden, the Netherlands) was carried out according to the manufacturer’s instruction. Light emission, resulting from the luciferase activity was measured for 10 seconds per well using an Infinite^®^ 200 PRO microplate reader (Tecan, Mechelen, Belgium).

### Data analysis

In the receptor activation bioassay, the activity of a peptide on TcSKR was represented by the increased luminescence units, calculated with the following formula: activity_peptide_ = (luminescence_peptide_ – luminescence_medium_)/luminescence_medium_ × 100. The activity of SK-related peptide was compared to that of nsSK and medium control by one-way analysis of variance with the Dunnett’s posttest in GraphPad Prism version 5.00 (GraphPad Software, La Jolla, CA). The activity of a SK-related peptide was also related to the activity of nsSK as a percentage value, calculated with the formula: relative activity of peptide (% nsSK) = activity_peptide_/activity_nsSK_ × 100.

## Additional Information

**How to cite this article**: Yu, N. *et al.* Flexibility and extracellular opening determine the interaction between ligands and insect sulfakinin receptors. *Sci. Rep.* **5**, 12627; doi: 10.1038/srep12627 (2015).

## Supplementary Material

Supplementary Information

## Figures and Tables

**Table 1:** Bioactivity of sulfakinin and sulfakinin-related peptides on the two *Tribolium castaneum* sulfakinin receptors (TcSKR1 and TcSKR2) in Sf9 cell-based reporter system.

Peptide	Sequence	relative activity (% nsSK)#	
		TcSKR1	TcSKR2
nsSK	FDDYGHMRF-NH_2_	100.0 ± 0.0*	100.0 ± 0.0*
2003	FDDYGHMRA-NH_2_	−2.0 ± 3.4	−3.5 ± 5.9
2004	FDDYGHMAF-NH_2_	58.0 ± 22.0	18.7 ± 10.2
2005	FDDYGHARF-NH_2_	−0.8 ± 4.4	11.9 ± 2.7
2006	FDDYGAMRF-NH_2_	0.5 ± 0.7	4.0 ± 9.9
2007	FDDYAHMRF-NH_2_	55.0 ± 7.1	8.5 ± 5.3
2008	FDDAGHMRF-NH_2_	5.3 ± 1.5	21.5 ± 10.7
2009	DDYGHMRF-NH_2_	7.8 ± 11.8	1.2 ± 13.7
2010	DYGHMRF-NH_2_	28.1 ± 15.6	8.6 ± 9.1
2011	YGHMRF-NH_2_	2.8 ± 2.5	11.7 ± 13.5
2053	GHMRF-NH_2_	0.6 ± 0.1	−4.8 ± 0.1
2052	HMRF-NH_2_	6.8 ± 0.2	1.8 ± 0.1
2051	MRF-NH_2_	4.4 ± 12.0	8.3 ± 6.6
1835	SDDY(SO_3_H)GHMRF-NH_2_	76.5 ± 7.6*	645.7 ± 126.6*
1591-1	EADY(SO_3_H)GHNleRF-NH_2_	82.6 ± 2.0*	113.7 ± 60.5*
1658	DDY(SO_3_H)GHMRF-NH_2_	64.8 ± 8.4*	516.8 ± 50.6*

^#^Relative bioactivity of a peptide calculated at concentration of 10 μM and related to the activity of nonsulfated SK (nsSK) peptide. Data are represented as mean ± SEM from at least two biological replicates.

^*^A peptide is active on the sulfakinin receptor (SKR).

**Table 2:** Summary of residues from TcSKR1 and TcSKR2 that are involved in the binding of sulfakinin peptides through polar interactions.

Peptide#	Sequence	Amino acid*	TcSKR1		TcSKR2	
			Region&	Amino acid*	Region&	Amino acid*
sSK	FDDY(SO_3_H)GHMRF-NH_2_	F1	—	—	ECL2	W194
		H4	TM VI	Y438	ECL2	K189
		Y6	TM V	D221	—	—
			ECL 2	E206	—	—
		D7	TM V	N217	—	—
		D8	TM V	N217	—	—
		F9	—	—	TM VII	K351
nsSK	FDDYGHMRF-NH_2_	F1	ECL 2	R199	ECL 2	E200
		H4	TM VI	Y438	ECL 2	R191
		Y6	ECL 2	E206	—	—
		D7	TM V	N217	ECL 3	S342
		D8	ECL 2	W207	—	—

^#^sSK, sulfated sulfakinin; nsSK, nonsulfated sulfakinin.

^*^Amino acid is represented with the single-letter code. The following numerical indicates the position from the N-terminus of a peptide or protein.

^&^TM, transmembrane region; ECL, extracellular loop. –no interaction detected.
