# Supplementary material for: Flexibility and extracellular opening determine the interaction between ligands and insect sulfakinin receptors
Source: Sci Rep. 2015 Aug 12;5:12627. doi: 10.1038/srep12627 (PMC4542541; doi:10.1038/srep12627)
Supplement: Supplementary Information [file srep12627-s1.pdf]

Supplementary materials to:

Flexibility and extracellular opening determine the interaction between ligands and insect sulfakinin receptors

Na Yu [1†], Moises João Zotti [2†], Freja Scheys [1], Antônio S.K. Braz [3], Pedro H.C. Penna [3], Ronald J. Nachman [4], Guy Smagghe [1*]

[1] Department of Crop Protection, Faculty of Bioscience Engineering, Ghent University, 9000 Ghent, Belgium

[2] Molecular Entomology and Applied Bioinformatics, Department of Crop Protection, Federal University of Pelotas, 96010-900, Pelotas, RS, Brazil

[3] Laboratory of Computational Biology and Bioinformatics, Federal University of ABC, 09210-170 Santo André, Brazil

[4] Insect Control and Cotton Disease Research Unit, Southern Plains Agricultural Research Center, USDA, College Station, TX 77845, USA .

† These authors contributed equally to this work.

* Corresponding author: Guy Smagghe

Email: guy.smagghe@ugent.be; Tel.: +32 92646150; Fax: +32 92646239

Supplementary material

Supplementary table 1. Residues in TcSKR1 and TcSKR2 involved in the binding of SK-related peptides through polar interactions.

Supplementary figure 1. Molecular dynamics of SKRs embedded in plasma membrane. Rectangular box containing SKRs, membrane, peptides and waters (Figs A-D). SKR1 with empty cavity, with docked nsSK and sSK (Figs A, B and C, respectively). Fig D depicts SKR2 with empty cavity. The line graph was constructed using RMSD from each protein complex over 120 ns (E). Lines colored differentially indicate the legend and letter in brackets corresponds to the complex used. The lines are colored as follows: SKR1 gray, SKR1_nsSK orange, SKR1_sSK red and SKR2 blue.

Supplementary figure 2. Ramachandran plots for SKR1 and SKR2. The contours indicated by yellow line indicate favored regions. The upper left regions highlighted by red contours indicated most favored regions for beta-sheet while bottom left indicated most favored regions for right-handed alpha helix. The upper right region indicated left-handed alpha helix. For SKR1 95.4% of residues are located in favoured regions while 3.4% in allowed region and only 1.1% in outlier regions. For SKR2 96.3% of residues are locate in favored regions while 3.4% in allowed regions and 0.3% in outlier region.

Supplementary table 1. Residues in TcSKR1 and TcSKR2 involved in the binding of SK-related peptides through polar interactions.

Peptide	Sequence	Amino acid*	TcSKR1		TcSKR2	
			Region [&]	Amino acid*	Region [&]	Amino acid*
2003	FDDYGHMRA-NH_2	A1	ECL 3	S451	TM 4	S178
					ECL 2	K184
					ECL 1	E109
		G5	TM VII	Q458	ECL 2	R191
				Q458		
		Y6	-	-	ECL 2	R191
		F9	TM V	L220	-	-
2004	FDDYGHMAF-NH_2	F1	TM IV	S184	-	-
		H4	TM V	D221	-	-
		Y6	ECL 2	E206	ECL 2	Y199
		D7	TM II	Q113	-	-
		D8	-	-	ECL 3	Y350
2005	FDDYGHARF-NH_2	F1	-	-	ECL 2	R191
		G5	ECL 2	E205	-	-
		Y6	-	-	ECL 3	S341
		D7	TM VII	Q458	ECL 2	K201
		F9	TM V	D221	ECL 2	K201
2006	FDDYGAMRF-NH_2	F1	-	-	ECL 2	K184
		R2	ECL 2	E213	-	-
		Y6	ECL 2	E206	-	-
		D7	ECL 2	S211	-	-
			TM V	N217	-	-
		D8	ECL 2	E205	-	-
		F9	TM V	D221	ECL 3	N346
						N347
2007	FDDYAHMRF-NH_2	F1	TM III	Q132	ECL 2	Y199
		M3	-	-	ECL 2	Y199
		H4	TM VII	Q458	-	-
		A5	-	-	ECL 2	K189
		F9	ECL2	Q214	-	-
2008	FDDAGHMRF-NH_2	G5	-	-	ECL 2	R191
		D7	TM VII	Q458	-	-
		D8	TM III	Q132	-	-
2009	DDYGHMRF-NH_2	F1	TM VI	W428	-	-
				H432	-	-
		R2	TM VII	S465	-	-
		H4	-	-	ECL 3	S341
		G5	ECL 2	C203	-	-

		D7	ECL 2	K202	-	-
		D8	ECL 2	E206	-	-
2010	DYGHMRF-NH_2	R2	-	-	ECL 2	S341
		H4	ECL2	E205	-	-
		Y6	TM V	N217		
				D221		
2011	YGHMRF-NH_2	H4	ECL 2	E206	ECL 2	R191
		G5	-	-	ECL 2	K201
		Y6	TM V	N217		
				N217		
2053	GHMRF-NH_2	F1	-	-	ECL 2	K201
		R2	TM VI	Y438	ECL 3	S341
		M3	TM V	N217		
		H4	TM V	N217		
2052	HMRF-NH_2	F1	TM VII	Q458	ECL 2	Y204
		H4	TM V	D221	ECL 3	Y350
				D221		
2076	FDDYGHMR-NH_2	R1	TM V	D221	ECL 1	H104
					ECL 2	R191
		H3	TM III	Q132	ECL 3	Y350
			ECL 2	E205		
		Y6	-	-	ECL 2	K201

*, amino acid is represented with the single-letter code. The following numerical indicates the position from the N-terminus of a peptide or protein.

[&], TM, transmembrane region; ECL, extracellular loop.

-, no interaction detected.

Supplementary figure 1.

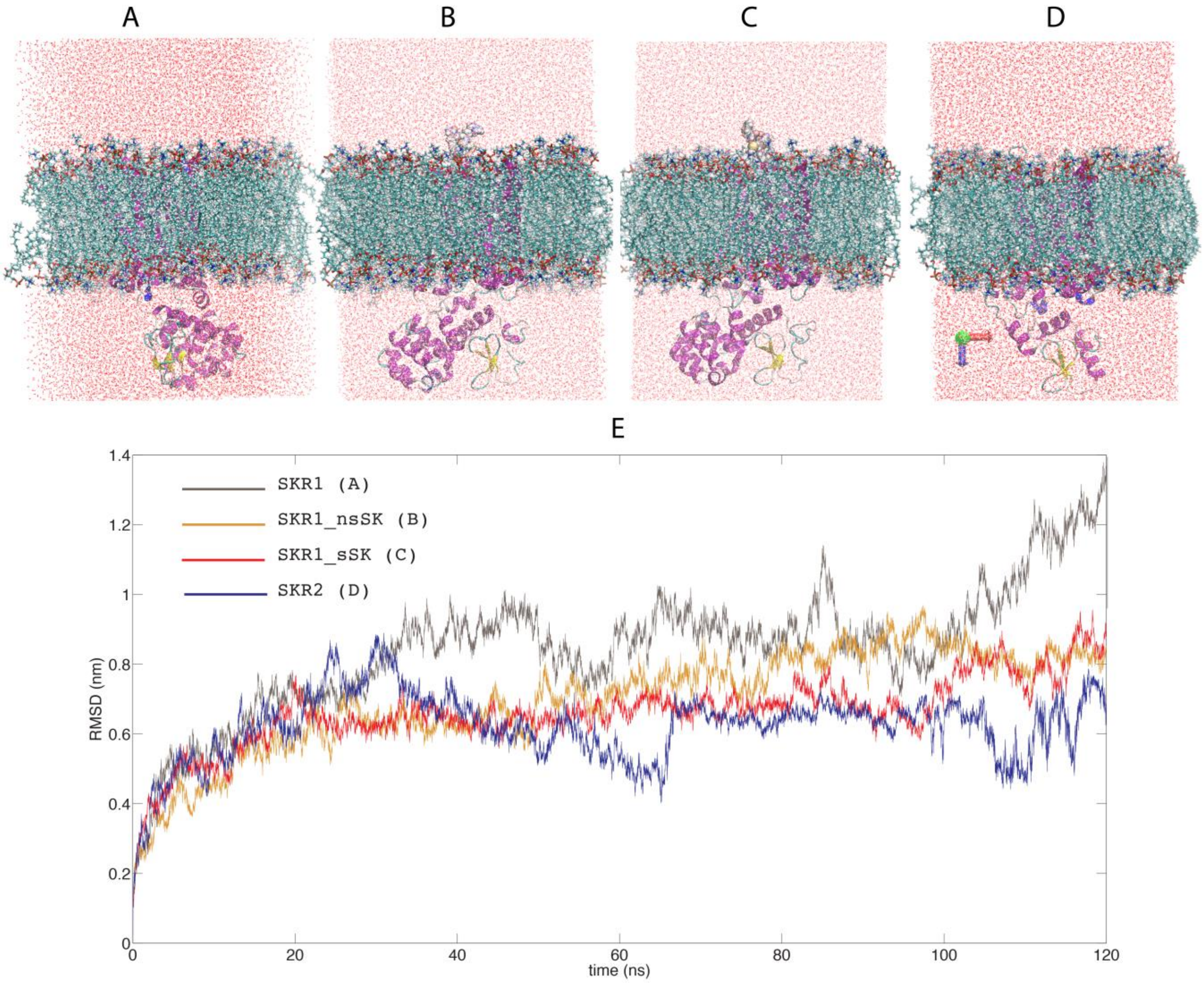

Supplementary figure 2

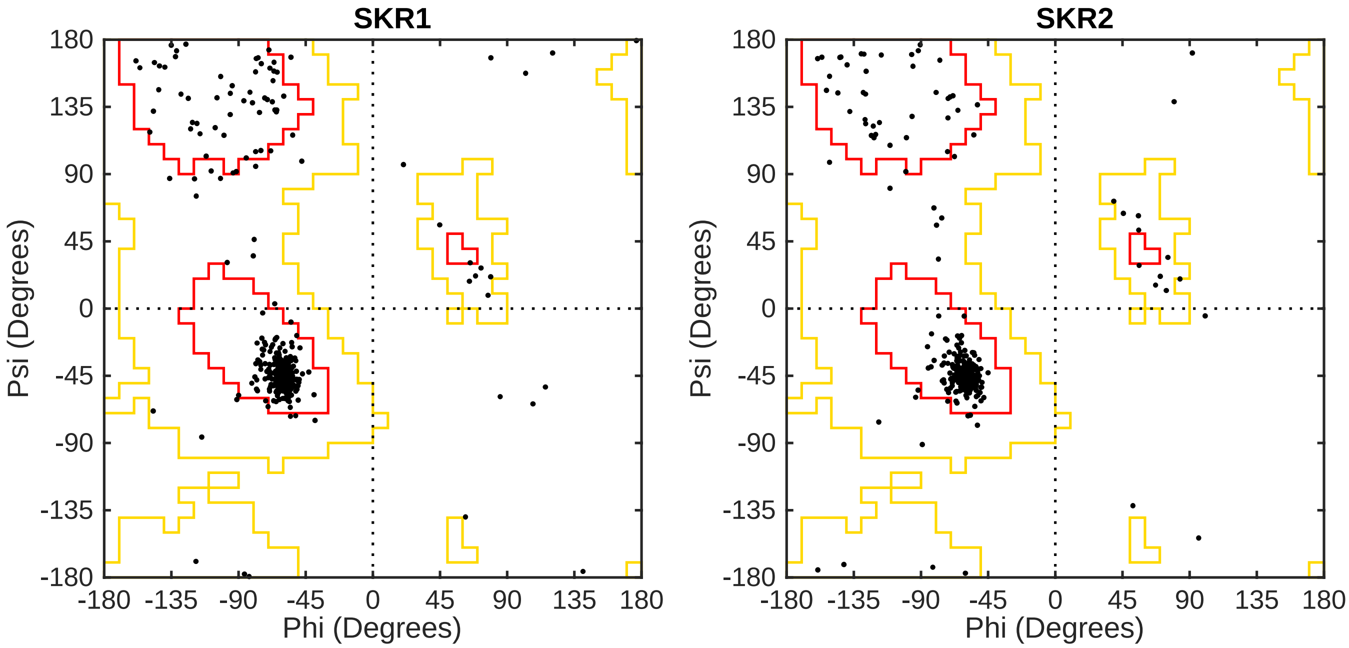

Evaluation of residues SKR1

Number of residues in favoured region (~98.0% expected):	418 (95.4%)
Number of residues in allowed region (~2.0% expected):	15 (3.4%)
Number of residues in outlier region :	5 (1.1%)

Evaluation of residues SKR2

Number of residues in favoured region (~98.0% expected) :	337 (96.3%)
Number of residues in allowed region (~2.0% expected) :	12 (3.4%)
Number of residues in outlier region :	1 (0.3%)
